# Imaginary Companions in Children with Autism Spectrum Disorder

**DOI:** 10.1007/s10803-018-3540-y

**Published:** 2018-03-21

**Authors:** Paige E. Davis, Haley Simon, Elizabeth Meins, Diana L. Robins

**Affiliations:** 1 Psychology Department, University of Huddersfield, EKG/09, Queensgate, Huddersfield, HD1 3DH UK; 2 Psychology Department, Drexel University, 3141 Chestnut St., Philadelphia, PA 19104 USA; 3 Psychology Department, University of York, Heslington, York, YO10 5DD UK; 4 AJ Drexel Autism Institute, 3020 Market St., Philadelphia, PA 19104 USA

**Keywords:** Autism spectrum disorder, Imagination, Imaginary companions, Social attribution, Social development

## Abstract

One of the deficits observed in autism spectrum disorder (ASD) is impaired imaginative play. One form of imaginative play common in many typically developing (TD) children is having an imaginary companion (IC). The occurrence of ICs has not been investigated extensively in children with ASD. We examined differences in parent report of IC between TD and ASD populations in 215 (111 with ASD) gender-matched children aged between 2 and 8 years. Findings indicate that significantly fewer children with ASD created ICs, although there were many between-group similarities in IC forms and functions. Results are discussed in terms of qualitative differences in play, social attributions, and how children with ASD conceptualize their ICs’ minds.

**Electronic supplementary material:**

The online version of this article (10.1007/s10803-018-3540-y) contains supplementary material, which is available to authorized users.

## Introduction

Imaginary or pretend play is an activity that typically developing (TD) children engage in frequently and spontaneously. However, children diagnosed with an autism spectrum disorder (ASD) show deficits in this behavior. Early in the ASD literature, Wing and Gould (1979) proposed that children with ASD had marked impairments in imagination, along with socialization and communication deficits. The issues with imagination that children with ASD have are so prevalent that one of the components of the diagnostic criteria is difficulty in sharing imaginative play (APA 2013; Jarrold 2003; WHO 1992).

Past research on imaginative behavior in children diagnosed with ASD shows that this population is profoundly delayed in imaginative play (Baron-Cohen 1987; Rutherford et al. 2007; Wolfberg et al. 2012), specifically social imaginative play (Lewis and Boucher 1988; Ten Eycke and Müller 2015). The generativity of pretend play is also slower for children with ASD than TD or language matched peers with learning disabilities (Jarrold et al. 1996). Pretend play enables children to engage in and enact different social roles (Howes 1988; Singer and Singer 1990), practice different social interactions using an appropriate script for that character or individual (Harris 2000), and develop an understanding of how social rules operate by using skills such as sharing, taking turns, or verbally interacting (Bruder and Chen 2007). Thus, higher levels of social pretend play are related to higher levels of peer oriented social competence (Uren and Stagnitti 2009).

The scarcity of pretend play in children with ASD therefore reduces opportunities for them to engage in imaginative play as a social activity (Wolfberg et al. 2012).

In order to explore imagination in children diagnosed with ASD, some researchers have focused on differences in children’s abilities to generate hypotheticals, or novel ideas, using Karmiloff-Smith’s (1990) “draw an impossible person” task (Low et al. 2009; Scott and Baron-Cohen 1996; Ten Eycke and Müller 2015). Children with ASD showed deficits in drawing impossible (imaginary) entities compared to both TD children and children with intellectual disability (Scott and Baron-Cohen 1996), after accounting for differences in executive functioning and generativity. In a similar paradigm, ASD and TD children were told to imagine a person walking in a “magic” door and coming out changed into a funny and strange looking being (Ten Eycke and Müller 2015). This script was used to increase the children’s comprehension of the request. They were then asked to draw the person and subsequently rated on the imaginative content of their drawing by two independent TD coders using proportional scoring criteria established by Marsh et al. (1996). Children with ASD created significantly less imaginative drawings. Interestingly, this imaginative deficit may be restricted to social content; when children with ASD drew both an impossible person and an impossible house (Ten Eycke and Müller 2015), only the person drawings were significantly less imaginative compared to TD controls. Ten Eycke and Müller theorized impoverished social experiences might lead to less social information processing, leading to the less elaborate forms of mental representation much like the early social attention negative feedback loop proposed by Dawson et al. (2004).

There are also other theories as to why children diagnosed with ASD might have impairments in social imagination behavior. Baron-Cohen (1987) posited that children with ASD may be unable to use second order representations or meta-representation, hence their difficulties in passing false belief tasks, and this could carry over to the ability to represent in social play situations. Deficits in play may be restricted to play that requires meta-representation (Leslie 1994), given that functional play may be intact in ASD.

One imaginative behavior that incorporates invisible and impossible entities and requires meta-representational ability is the creation of imaginary companions (ICs) (Singer and Singer 1990; Taylor 1999). IC creation is seen in up to 50% of TD children (Gleason and Hohmann 2006; Motoshima et al. 2014). An IC according to Svendsen (1934) is “an invisible character named and referred to in conversation with other persons or played with directly for a period of time, at least several months, having an air of reality for the child, but no apparent objective basis” (p. 988). Svendsen did not include personified objects (POs), toys that a child plays with for extended periods of time, but more recent definitions consider them to be the equivalent of having an IC as long as they have a stable personality (Taylor 1999).

There are several factors that relate to the probability of IC creation in TD children. Youngsters between the ages of 3 and 6 years are most likely to engage with an IC (Singer and Singer 1990). It is also more probable for first-born children to play with these imaginary creatures than children with siblings (Bouldin and Pratt 1999; Manosevitz et al. 1973). Finally, girls are more likely than boys to develop ICs (Pearson et al. 2001; Carlson and Taylor 2005; Gleason and Hohmann 2006), although this may relate to cultural factors and gender normativity (Taylor 1999).

Children who create ICs have been shown to have advantages compared to children without these entities (NIC). Some studies report that having an IC is related to superior ToM and emotion understanding abilities (Giménez-Dasi et al. 2016; Taylor and Carlson 1997), although others have reported null findings for this relation (Davis et al. 2011, 2014; Fernyhough et al. 2007). Children with ICs are more likely than their NIC counterparts (a) to know that their minds are opaque to others (Davis et al. 2011), (b) to form richer narratives when storytelling (Trionfi and Reese 2009), (c) show more sophisticated self-directed speech (Davis et al. 2013), and (d) are better able to take the listener’s perspective into account during a referential communication paradigm (Roby and Kidd 2008). They also are more likely to describe friends with reference to their mental characteristics instead of behavioral tendencies or physical appearance (Davis et al. 2014). Children with ICs also give these friends the same status as real friends, using ICs as a way to avoid loneliness in some instances (Bouldin and Pratt 1999).

There is a paucity of scientific research on IC creation by children with ASD. Given that children with ASD have imagination deficits paired with ToM impairment, it would seem that these children would not be strong candidates for creating ICs. Many of the cognitive advantages explored in TD children with ICs such as superior ToM, emotional understanding, and social communication ability are in the same domains as impairments seen in children with ASD. Even the fact that girls are more likely to play with ICs whereas children diagnosed with ASD are more typically boys reduces the odds that a child with ASD would create one of these friends. However, because children with ASD were able to complete the Karmiloff-Smith (1990) “draw an impossible person task” and partake in creating an impossible being, it seems plausible that some children in the population could have the means to create an IC spontaneously.

Furthermore, ICs have also been shown to have different types of functions depending on their child creator (Bouldin and Pratt 1999). For example, children with limited social interactions will sometimes create ICs to talk with when they have no one else to relieve loneliness. Children with ASD often have less social interaction than their peers, so this might be another indicator that it could be possible for these children to create an IC on their own (Bauminger et al. 2003). There have been parental accounts on internet chat boards of children with ASD creating ICs, as well as scholarly evidence indicating that children with ASD create ICs (Calver 2009).

The present study aimed to discover whether children with ASD spontaneously create their own ICs, and whether the form of IC (completely invisible or personified in an object, the complexity of the IC’s characterization) differed between ASD and TD children. We were also interested in investigating whether the ASD and TD groups differed with respect to the reported function of the IC or the age at which ICs were created. In addition, we explored factors relating to IC creation within the ASD group, investigating whether age at which children were diagnosed with ASD or children’s reported cognitive functioning related to the tendency to create an IC. Finally, the present study asked whether certain functions of ICs may be congruent with the needs (e.g., social communication) of children with ASD.

## Method

### Participants

Participants were 111 parents of children with ASD (see Table 1) between 24- and 96- months old (*M* = 59.10 months, *SD* = 27.25); 79% (n = 88) were boys. The sample consisted of 85.6% Caucasians, 4.5% African Americans, 2.7% Asian, 3.6% Biracial, and 3.6% preferred not to answer. Information on economic status was not collected on this group. Parents reported that their children were diagnosed with ASD between 10 and 96 months of age (*M* = 43.05, *SD* = 22.52). All diagnostic variables can be found in Table 1.

**Table 1:** Parental indication of child diagnoses

Diagnosis	N	Percentage indicating yes (%)^a^
Autism	49	44.1
Autistic disorder	5	4.5
Autism spectrum disorder	50	45
Asperger’s disorder	21	18.9
Pervasive developmental disorder not otherwise specified	33	29.7
Other	18	16.2
Level of functioning		
High	46	41
Low	4	3.6

*Note Classifications or descriptions of were not mutually exclusive, and parents were asked to check all that apply

^a^The remainder of the parents did not report a level of functioning

A second, previously existing data set of economically diverse parents of typically developing children reporting on their child’s IC status in a lab setting was used to compare the two groups. Participants were randomly matched with the ASD group based upon gender resulting in a TD group of 104 parents of children (81 boys) aged between 59- and 64- months (*M* = 61.27, *SD* = 1.14). Only 81 boys participated, so seven boys were not matched. University ethics committees approved both protocols and all parents gave full informed consent.

### Materials and Procedures

Parents of children with ASD were recruited through materials stating that the researchers wanted to investigate how children with ASD think. Materials were not directed at parents that might be prone to have a child with an IC to provide a better, less biased picture of how many children in this population create ICs. Parents were recruited via (1) emails to listservs for families of children with ASD, (2) emails to agencies that had local, state, and national outreach, (3) flyers posed on social media, and (4) flyers handed out at different regional events. Parents then completed an online questionnaire through Qualtrics. Parents were provided with a link that they could access at their own computer or on their phone. The questionnaire took anywhere from 3 to 20 min to fill out depending on whether the parent reported that their child had created an IC.

Parents of typically developing children were participating in a larger ongoing longitudinal study of which the IC questionnaire was a part. They completed a variation of this questionnaire in person on paper at a lab. They did not complete the section on demographics, diagnostics, and education at the time they were given the questionnaire. The last seven free-response questions about ICs were also not given to these parents.

### The Questionnaire

Parents of children with ASD first completed four forced-choice questions about demographics: age, sex, race, and ethnicity. These questions were followed by three forced-choice questions about the child’s educational environment: whether they were publicly or privately educated, if they had individualized education plans, the type of school programs they attended, and a free response item to clarify school situation. Finally, they answered three forced-choice questions about the child’s diagnoses, checking all labels that applied, including a high or low functioning option, and “other” which allowed them to type in additional diagnoses, the type of professional who provided the diagnoses, and the age at time of an ASD diagnosis.

Next, parents were asked if their child had ever created an IC. This is the point where TD group began their paper questionnaire. All parents were asked, *does your child have an imaginary friend or have they had one in the past? (This could be a completely imaginary friend or a toy or stuffed animal that your child has played with for over three months and has a stable personality)*. If parents of children with ASD did not report their child having an IC, they were thanked for participating and the questionnaire closed. If the parent responded affirmatively, they were taken to an adaptation of Taylor and Carlson’s (1997) IC interview. All parents were asked about whether the IC was a doll/toy, or completely invisible, the IC’s name, when their child created the IC, the IC’s gender, age, and appearance, what the child enjoys and dislikes about the IC, and where the IC lives and sleeps. Parents of TD children ended at these questions, and 24 of the 47 (51%) did not choose to answer the more elaborate IC questions in this part of the larger longitudinal study. Only two of the 18 (11%) parents of the children with ASD failed to report more on their child’s IC. The online questionnaire for parents of children with ASD also asked free response questions about activities the child and IC participate in together, what they talk about, how and when the child stopped playing with the IC, whether the child uses the IC to communicate needs, if the IC has any unique qualities. ICs were classified as completely invisible (iIC) or personified in an object (PO); examples of iICs and POs can be found in Table 2.

**Table 2:** Examples of imaginary companions in children diagnosed with ASD

IC	Ghosty Bubble: an invisible bubble person who was fun to talk to and slept on a bubble bed next to the child. When the child wanted to be alone he could be popped
IC	Pretend Ada: An invisible version of a child’s school friend who plays with the child when she needs a friend
IC	Mikey: An invisible ninja who lives in the sewer and is played with daily and read to by the child
IC	Andrew: An invisible boy who drives a rainbow colored Lincoln and sleeps on a bunk bed
PO	Hatch: A stuffed chicken that the child carries around and uses in stop motion films
PO	Teddy: A stuffed bear used to help the child sleep and makes sure he has good dreams
PO	Batman: A toy that the child plays with and likes because Batman helps people that are in trouble

### Social Attribution Coding

Parent report on the open-ended questions where parents were asked to describe various features of their child’s IC was coded using a variation of Klin’s (2000) animation index for social attribution. This index was initially developed to measure the level of social attribution children with ASD ascribe to geometric shapes enacting a social story, and was adapted to ascertain the level of social attribution parents reported when describing their children’s ICs. Descriptions were placed into 3 exclusive and exhaustive categories of attribution: (1) behaviors (e.g., playing with the IC, or bossing the IC around); (2) cognition, intention, and motivation (e.g., the IC being kind, or naughty); and (3) relationships and personality traits (e.g. ‘the pretend version of a real girl’, ‘she is his baby’). Each category contained levels of sophistication, so, the child’s score for social attribution to their IC would increase with greater attributive sophistication. Raw scores for each parent response were summed to a composite score, and divided by the total number of descriptions, yielding a percentage score (accounting for variability in the number of responses provided).

### Qualitative Analyses

Open-ended IC questions were analyzed using grounded theory, which searches for prominent themes running throughout the interviews and naturally arising in the parent’s answers about their child’s ICs. Both ASD and TD populations were examined.

## Results

### Descriptive Statistics and Preliminary Analysis

Based on parent report, 16.2% of children diagnosed with ASD were reported as having created ICs. Of the children creating ICs, 38.8% were iICs and 61.1% were POs, such as a stuffed toy or doll. Examples of these can be seen in Table 2. Of the TD population, 45.2% of the children were reported to have an IC. For this group, 70.2% were iICs and 29.8% were POs. Child gender in the ASD group was not significantly related to IC status, χ^2^(1) = 1.21, *p* = .272, *V* = .10. There was also no association between gender and IC status in the TD population χ^2^(1) = 1.53, *p* = .216, *V* = .12. Age was not related to whether or not children created ICs in the ASD *F*(1,93) = 0.53, *p* = .585, η^2^ = .012, or TD, *F*(1,87) = 0.06, *p* = .814, η^2^ = .001, populations.

### Differences in IC Creation Between ASD and TD Children

Chi square analyses were used to investigate whether children diagnosed with ASD were as likely as their TD counterparts to create ICs. IC creation was significantly less common in the ASD sample, χ^2^(1) = 21.37, *p* < .001, *V* = .32. When only looking at the differences between iIC and PO creation, children in the ASD sample were less likely than the TD children to create iICs η^2^(1) = 5.40, *p* = .020, *V* = .29.

Group differences in the age parents reported their child beginning to play with their IC were investigated using a one way ANOVA. Children with ASD began playing with ICs significantly later than TD children according to their parents, *F*(1,35) = 5.09, *p* = .031, η^2^ = .130. Age of first parent reported IC interaction examining group differences between iIC and PO creation was investigated in children with ASD and then in TD children in an ANOVA. No effect was found for the type of IC in ASD, *F*(1,16) = 0.19, *p* = .672, η^2^ = .012; or TD populations, *F*(1,18) = 0.07, *p* = .800, η^2^ = .004. Means and standard deviations for age related variables can be found in Table 3.

**Table 3:** Mean (standard deviation) scores as a function of group and IC status

	Group	N^a^	TD children	N^a^	ASD children
Current age (months)	IC	29	61.34 (1.23)	5	60.00 (16.97)
	PO	14	61.21 (0.89)	9	68.00 (27.50)
	NIC	45	61.24 (1.17)	79	58.03 (27.80)
Age of IC creation (months)	IC	13	21.77 (8.70)	6	32.00 (14.53)
	PO	6	23.17 (15.16)	11	37.09 (26.54)
Age of ASD diagnosis (months)	IC		–	7	37.86 (17.61)
	PO		–	9	25.67 (8.09)
	NIC		–	83	45.37 (23.16)
Raw attribution scores (all descriptions including social attributions)	IC	14	2.43 (2.24)	7	3.71 (2.75)
	PO	7	3.14 (3.29)	11	1.82 (2.71)
	Total	20	2.80 (2.57)	18	2.56 (2.81)
Social attribution scores (raw scores divided by total descriptions)	IC	14	49.23 (38.98)	7	55.00 (32.62)
	PO	7	61.19 (59.99)	11	26.55 (35.24)
	Total	20	53.22 (45.83)	18	37.61 (36.19)

*Note all ages are reported in months. Social attribution scores are raw scores divided by total number of parent descriptions. Social attribution total scores exceeded the number of parent descriptions in four cases, so percentages were over 100. Standard deviations are given in parentheses

^a^There are missing data for some columns. The N reflects the number of parents that reported for specific variables

### Social Attribution Analysis

Parental reports on IC descriptions and interactions were coded to reflect social attributions ascribed to the IC by their children. The data for the ASD and TD groups are presented in Table 3. There was no difference in social attributes between the TD and ASD groups, *t*(37) = 1.36, *p* = .251, *d* = .38. There were also no differences in social attributions attributed in children with iICs versus POs in either the ASD, *t*(16) = 1.72, *p* = .105, *d* = .84 or TD groups, *t*(19) = − 0.55, *p* = .586, *d* = .24. Means and standard deviations for social attribution variables can be found in Table 3.

### IC Form and Function Analysis

The IC’s gender was then analyzed to determine if there were group differences in children interacting with a male, female, or non-gendered IC. No difference was found between the TD and ASD groups, χ^2^(2) = 1.34, *p* = .512, *V* = .18. Parents were also asked if their child dislikes anything about their IC. Significantly more parents in the TD group reported their children disliking things about their IC than in the ASD population *t*(34) = 3.69, *p* = .002 (see Table 4).

**Table 4:** Frequencies and (percentages) of IC functions

	Group	TD children N (%)	Children with ASD N (%)
Gender^a^	Male	13 (59.1%)	9 (50%)
	Female	4 (18.2%)	2 (11.1%)
	Non-gendered	5 (22.7%)	7 (38.9%)
IC dislikes	No dislikes	10 (55.6%)	18 (100%)
	Dislikes	8 (44.4%)	0 (0%)
Main function themes	Social	12 (52.2%)	7 (43.8%)
	Comfort	4 (17.4%)	5 (31.3%)
	Neither social nor comfort	7 (30%)	4 (25%)

*Note this table consists solely of frequencies followed by within-group percentages in parentheses. Percentages reflect total responses given not including missing data

^a^The non-gendered variable refers to parents reporting that the IC had no gender or that a child had more than one IC with more than one gender

### IC Form and Function Thematic Analyses

**Fig. 1:**
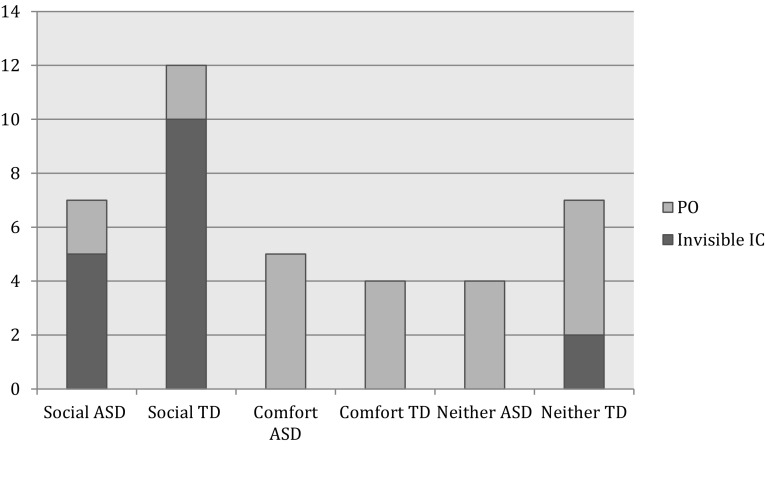
Functions of invisible imaginary companions iICs and personified objects POs in children with autism spectrum disorder ASD and typically developing TD children (n = 39 ICs)

Parent description of their child’s ICs were then analyzed to look further into the forms and functions that these entities represented in the population of children with ASD. The two main themes of social support or comfort were found running through the questionnaires related to the function of the child’s IC. Table 5 shows examples of parent responses to the functionality their child’s IC. Parent reports were then grouped into three categories of function (Social, Comfort, and neither social nor comfort). Figure 1 shows the parent report of functions of iICs and POs in children in the TD and ASD populations. Fisher’s exact test indicated no significant group difference in children’s functional use of their IC for social or comfort purposes (*p* = .432).

**Table 5:** Examples of social and comfort descriptions

Description type	Parent description of IC
Social descriptions	He’s fun to talk to
	Sometimes they talk about huge plans to take over the world and have things their way
	She will play with her for days
Comfort descriptions	He uses it for comfort, sleeps with it, and brings it everywhere he goes
	She is cuddly, and sleeps in his bed. She is his baby
	It helps him sleep better and keeps away bad dreams

### Variables Related to ICs in Children Diagnosed with ASD

A binomial logistic regression was used to investigate whether the age of first diagnosis related to the likelihood of a child creating an IC. The regression model was statistically significant χ^*2*^(1) = 6.74, *p* = .009. The model explained 11.2% (Nagelkerke *R*^2^) of the variance. Children’s age of diagnosis was further investigated by looking at differences in age of diagnosis between iIC, NIC, and PO children in an ANOVA finding a main effect of group *F*(1,98) = 3.47, *p* = .035, η^2^ = .067. Post-hoc analyses indicated that children creating POs were diagnosed at a significantly younger age than NIC children (*p* = .037), however there were no differences in age of diagnosis between iIC and PO children (*p* = .822), or iIC and NIC children (*p* = 1.000). All age-related variables can be found in Table 3. Using Fisher’s Exact test, there were no differences in IC creation between the high and low functioning children (*p* = .603).

Parents of children diagnosed with ASD were also asked whether their child uses the IC to communicate needs. Of the 17 parents reporting a child with an IC, three (17.6%, 1iIC and two POs) of the parents responded that their child did speak through the IC to communicate their needs, whereas 13 (76.5%, five iICs and eight POs) responded that their child did not use the IC in this way, and one (5.9%, IC) was unsure.

## Discussion

This exploratory study’s main aim was to discover what proportion of children diagnosed with ASD created ICs, investigating potential differences in ICs both within the ASD group and in comparison with TD children. Our findings indicate that a substantial minority of children diagnosed with ASD (16.2%) do spontaneously create ICs, with similar features to TD children’s ICs in some respects (e.g., iIC social and PO comfort functions). Furthermore, ICs’ gender and their reported social attributions did not differ between groups. However, parents of children with ASD were less likely than those of TD children to indicate that their children had created an IC to their knowledge, and when parents did notice IC play, it was at a later age than TD parents. Furthermore, the type of IC (iIC or PO) differed in that, compared with their TD peers, the ASD sample created fewer iICs, and were less likely to talk to their parent about disliking parts of their IC’s personalities. Within group differences in children diagnosed with ASD revealed earlier diagnoses for children creating ICs and IC type.

Finding a subset of children with ASD who spontaneously create ICs is contrary to much of the research on imagination in children with ASD, and in some instances, even the diagnosis itself (APA 2013; Baron-Cohen 1987; Jarrold et al. 1996). However, the argument has been made that certain individuals presenting with ASD are very creative and imaginative (Roth 2007), based on examining art and literature created by ASD and TD individuals. Children who did create ICs created them at later age than their TD peers which is congruent with previous research on imagination in children with ASD (Rutherford et al. 2007; Wolfberg et al. 2012), however in line with Roth (2007), our findings showed that there were more similarities than differences in ICs between the ASD and TD groups, suggesting that this imaginative behavior is not necessarily affected in children diagnosed with ASD. Children in both groups created ICs and there were no gender differences in creation. Furthermore, many of the features of the ICs created by TD and ASD children were also similar. A similar degree of social attributions was reported to be given to the ICs of both groups, even when taking into account how many opportunities parents had to describe their child’s IC. These results are surprising, as past research on social attribution has found that even children with ASD who are higher functioning and can pass ToM tests have deficits in social cognition (Klin 2000). The similarities between parent descriptions of their children’s social attributions across the ASD and TD groups suggest that perhaps ICs among children with ASD serve socially comparable purposes to those of TD children. Although, since a higher proportion of parents in the ASD group completed the more in-depth IC questions, it also may be the case that the parents of children with ASD might be more responsive to surveys about their child’s inner lives.

The finding that children diagnosed with ASD were not reported to dislike things about their ICs as frequently as TD children suggests that the ICs that are created by children with ASD might have less well developed personalities and minds, due to documented problems in ASD children in conceptualizing other minds (Baron-Cohen et al. 1985). Alternatively, children diagnosed with ASD may be spontaneously creating ICs that are qualitatively different from those of TD children and may thus engage with them in qualitatively different ways, for example using their IC for comfort, social, or other purposes which have not been analyzed statistically due to the small sample size of the IC children. It has been shown that play often takes on different forms in ASD (Honey et al. 2007); perhaps IC play is another example of this phenomenon. A second possibility is that the parents of children with ASD and ICs may be more focused on the mental lives as well as behaviors of their children as a result of their diagnosis and emphasis put on monitoring their child’s symptoms, thus making them more likely to attend to their children’s interactions with their IC, as the lack of parent report in the TD population might suggest.

This parental attentiveness could also be a reason for the finding within the ASD group that age of diagnosis was related to IC status. Children who created POs were diagnosed significantly younger than children who did not have ICs; children who created iICs were not significantly different in age from the other two groups. There are several different reasons for children diagnosed earlier to create POs. It may be the case that children diagnosed younger had more access to early intervention, which promoted development of social engagement. In addition, many factors, including symptom presentation, early detection strategies used by the child’s healthcare provider, urban versus rural locale, and other sociodemographic variables relate to age of diagnosis (e.g., see Daniels and Mandell 2014 for review). Future research could investigate whether these sociodemographic variables relate to the type of IC created by children with ASD.

Other potential differences in ICs within the ASD group were investigated in relation to parent reports of whether their child was high or low functioning. Children reported to be high versus low functioning did not differ with respect to their tendency to create an IC. However only four parents reported their child as low functioning, despite the fact that 33 were reported to have an additional developmental disorder. It is therefore difficult to draw strong conclusions on the relation between functioning level and tendency to create an IC in children with ASD.

To look at the functions played by ICs in both TD and ASD populations, a thematic analysis was carried out on both groups of parent descriptions of their child’s IC. In terms of using an IC to communicate needs to a parent, only three (17.6%) of the children with ASD with ICs reportedly used their IC for communicative purposes. ICs have been shown to have many communicative functions in TD children (Hoff 2005), but communication was not probed in the TD questionnaire, so direct comparison is not possible. Thematic analysis identified two main functions of ICs: social and comfort purposes in both samples. The hypothesis made about the functions of ICs entailed the possible relief of loneliness, as this is reported as a typical function of the IC, and research highlights higher reported levels of loneliness in children with ASD (Bauminger et al. 2003; Hoff 2005). Alleviating loneliness was not, however, reported as a function of ICs in parents of children with ASD.

The results reported here should be interpreted in light of some limitations of the present study. All measures were assessed by parental report. Although parents of TD children have been shown to be the best at reporting the imaginative lives of their children (Gleason 2004), future research should include child report, as suggested by Taylor (1999). Parents may not be aware of the existence of an IC, and this may be particularly the case in parents of children with ASD, who may not be able to talk to or play with ICs as obviously as TD children. The finding that ICs were predominantly reported to be POs by parents in the ASD group is in line with this suggestion. Future research should therefore attempt to gather information on the existence of ICs in children with ASD from the children themselves, and should also assess autistic traits in the parents to explore whether these may help explain the lower reported frequency of ICs in children with ASD. Our findings open up some interesting avenues for future research into the relation between ICs and autism symptomology. For example, face-to-face research would enable children with ASD’s cognitive abilities to be assessed directly in order to investigate in greater depth how individual differences in functioning (e.g. social vs. comfort) and symptom severity in children with ASD relate to their creation of ICs.

A second limitation is that the group of ASD children reported to have ICs was small, meaning that the within-group analyses were lacking in power. The third issue is one of generalizability. The parents of children with ASD were recruited mainly from Facebook support groups and local ASD events, and is therefore not a representative sample. Finally, matching participants on age, and including a comparison group of children with developmental delay to allow for matching ASD children for cognitive functioning, would provide more information on which aspects of IC creation and play may be the unique to children diagnosed with ASD.

Broadly, our results confirm much of the research on deficits in ASD children’s spontaneous imagination behavior. However, the similarities identified between the ICs of TD children and those with ASD who had created ICs suggest that differences between TD and ASD children on this specific high-level imaginative behavior may be quantitative rather than qualitative.

## Electronic Supplementary material

Below is the link to the electronic supplementary material.

Supplementary material 1 (SAV 1371 KB)
